# Bariatric Surgery as a Molecular Modulator: The Role of FSHR Polymorphisms in Enhancing eNOS Expression and Reproductive Hormone Dynamics in Women with Severe Obesity

**DOI:** 10.3390/biomedicines13010067

**Published:** 2024-12-30

**Authors:** Charalampos Voros, Despoina Mavrogianni, Kyriakos Bananis, Alexios Karakasis, Anthi-Maria Papahliou, Vasileios Topalis, Antonia Varthaliti, Raphail Mantzioros, Panagiota Kondili, Menelaos Darlas, Regina Sotiropoulou, Diamantis Athanasiou, Dimitris Mathiopoulos, Panagiotis Antsaklis, Dimitrios Loutradis, Georgios Daskalakis

**Affiliations:** 1 1st Department of Obstetrics and Gynecology, ‘Alexandra’ General Hospital, National and Kapodistrian University of Athens, 80 Vasilissis Sofias Avenue, 11528 Athens, Greece; depy.mavrogianni@yahoo.com (D.M.); alexioskarakasis@gmail.com (A.K.); anthipapahliou@gmail.com (A.-M.P.); antonia.varthaliti@hotmail.com (A.V.); rafahsb@hotmail.com (R.M.); giotakondyli27@gmail.com (P.K.); mdarlas2110@gmail.com (M.D.); reginasotirop@gmail.com (R.S.); panosant@gmail.com (P.A.); gdaskalakis@yahoo.com (G.D.); 2 King’s College Hospitals NHS Foundation Trust, London SE5 9RS, UK; kyriakos.bananis@nhs.net; 3 Department of Internal Medicine, Hospital of Thun, 3600 Thun, Switzerland; vtopalismd@gmail.com; 4 IVF Athens Reproduction Center V. Athanasiou, 15123 Maroussi, Greece; diamathan16@gmail.com; 5 Rea Maternity Hospital S.A., Avenue Siggrou 383 & Pentelis 17, P. Faliro, 17564 Athens, Greece; dimitrismathiopoulos@gmail.com; 6 Fertility Institute-Assisted Reproduction Unit, Paster 15, 11528 Athens, Greece; loutradi@otenet.gr; 7 Athens Medical School, National and Kapodistrian University of Athens, 15772 Athens, Greece

**Keywords:** infertility, bariatric surgery, FSHR, Asn680Ser, ENOS, oxidate stress

## Abstract

Background/Objectives: Severe obesity (BMI > 40 kg/m^2^) has a severe influence on vascular health and reproduction. This study looks at how bariatric surgery affects endothelial nitric oxide synthase (eNOS) expression and reproductive hormone regulation across different follicle-stimulating hormone receptor (FSHR) polymorphism groups in women with extreme obesity. Methods: Twenty-nine women with extreme obesity had bariatric surgery. Pre- and post-surgery levels of eNOS and reproductive hormones such as follicle-stimulating hormone (FSH), sex hormone-binding globulin (SHBG), anti-Müllerian hormone (AMH), and antral follicle count (AFC) were assessed. Patients were divided into three FSHR polymorphism groups (Ser/Ser, Asn/Asn, and Ser/Asn), and results were compared between them. Statistical techniques were used to determine changes and relationships. Results: Bariatric surgery led to substantial increases in eNOS expression across all FSHR polymorphism groups (*p* < 0.0001), with the Ser/Ser group exhibiting the most variability. Prior to surgery, the Ser/Ser group had substantially higher FSH levels (7.41 ± 0.60 mIU/mL) than the Asn/Asn group (5.20 ± 0.63 mIU/mL, *p* < 0.001). Following surgery, FSH levels rose in the Ser/Ser group (9.45 ± 0.87 mIU/mL), with significant differences between the Ser/Ser and Ser/Asn groups (mean difference = 0.97, *p* = 0.019). SHBG levels had a negative connection with eNOS expression after surgery (r = −0.365, *p* = 0.049). AMH and AFC remained constant throughout polymorphism groups. BMI decreased uniformly, with an average of 15.2 ± 1.8 kg six months after surgery. Conclusions: Bariatric surgery improves vascular health and regulates reproductive hormones, especially in individuals with the Ser/Ser genotype. These findings indicate the possibility of combining genetic testing and bariatric therapies to improve infertility treatment in obese women.

## 1. Introduction

Obesity is a complicated metabolic disorder with far-reaching consequences for overall health and reproductive function, particularly in women. In the United States alone, more than 30% of the population is obese, with similar increases reported elsewhere, particularly in developed nations [1,2,3]. Obesity, caused by dietary and lifestyle changes such as excessive sugary beverage consumption and prolonged sedentary activity, is currently the second biggest cause of preventable mortality after smoking [4,5,6,7].

Obesity-induced reproductive dysfunction is molecularly linked to changes in endothelial nitric oxide synthase (eNOS) activity. eNOS generates nitric oxide (NO), a crucial regulator of vascular tone and cellular signaling that is required for follicular development, oocyte maturation, ovulation, and embryo implantation [8,9,10]. NO, a highly reactive and short-lived inorganic free radical, operates as a unique biological signaling molecule that affects a range of physiological and pathological processes [8,9]. However, in the setting of obesity, increasing oxidative stress depletes the essential eNOS cofactor tetrahydrobiopterin (BH4), causing eNOS “uncoupling”. This uncoupling causes the generation of superoxide (O_2_^−^) rather than NO, resulting in oxidative byproducts such as peroxynitrite (ONOO-), which induce cellular damage and limit NO bioavailability [11,12]. Elevated nitrite (NO_2_) and nitrate (NO_3_) levels indicate aberrant NO metabolism and decreased vascular function, which interferes with folliculogenesis, oocyte quality, and embryo implantation. These changes are also associated with polycystic ovarian syndrome (PCOS), which contributes to infertility [13,14].

Bariatric surgery is an effective treatment for extreme obesity, improving quality of life and lowering morbidity and death by addressing excess weight [15,16,17]. This surgical procedure also improves reproductive health by lowering testosterone and dehydroepiandrosterone sulfate (DHEA-S) levels while boosting luteinizing hormone (LH) and follicle-stimulating hormone (FSH) levels, thereby improving ovarian function. Furthermore, bariatric surgery has been demonstrated to restore eNOS activity, reduce oxidative stress, and boost NO bioavailability, all of which benefit vascular health and reproductive outcomes.

FSH regulates female reproductive physiology by interacting with its receptor, FSHR, which is mostly expressed in granulosa cells. This connection is necessary for follicular maturation, luteinization, and ovulation, which are all regulated by LH [18]. While FSH was generally assumed to act primarily on gonadal tissues, new research showed that its receptor was expressed in extragonadal tissues, including bone and adipose [19]. Genetic differences, such as single-nucleotide polymorphisms (SNPs) in gonadotropins and receptors, have a further impact on ovarian response and reproductive outcomes [20,21]. Among these, the *FSHR rs6166 (c.2039A>G, p.Asn680Ser)* polymorphism has been intensively investigated as a biomarker for ovarian responsiveness to FSH stimulation [22,23]. The *Asn/Asn* genotype is linked to normal FSH sensitivity, but the *Ser/Ser* genotype, despite being the wild type, has lower FSH receptor sensitivity. Women with the Ser/Ser genotype have greater serum FSH levels, a larger total dosage of gonadotropins during ovarian stimulation, and fewer retrieved oocytes [24,25]. These findings indicate that the Ser/Ser genotype may play an important role in tailored approaches to ovarian stimulation and fertility management.

In conclusion, the molecular basis of obesity-related reproductive dysfunction emphasizes the importance of eNOS and FSHR. Bariatric surgery is emerging as a viable option for not only weight loss but also for restoring eNOS activity and improving ovarian function. Understanding the interactions between obesity, biochemical pathways such as NO signaling, and genetic variants such as FSHR polymorphisms opens up new possibilities for improving reproductive health outcomes in affected individuals.

## 2. Materials and Methods

This study comprised 29 women under 40 with a BMI of 40 kg/m^2^ or above, as per the Helsinki Declaration’s ethical standards. The ethical permission was obtained by the Medical School of Athens (permission No. 48859, Date: 30 October 2020) and Alexandra General Hospital (Approval No. 4345, Date: 1 April 2023). All participants provided informed consent for this prospective study, which took place from March 2022 to November 2024 at the First Department of Obstetrics and Gynecology at “Alexandra” General Hospital. Infertility was defined as the inability to achieve clinical pregnancy after a year of regular, unprotected sexual intercourse.

From March 2022 to November 2024, participants were recruited from a single facility specializing in bariatric surgery and reproductive health. Endocrinologists, gynecologists, and obesity clinics provided referrals for recruitment. Eligibility was determined by lengthy clinical interviews, comprehensive physical examinations, and laboratory tests to establish infertility and metabolic conditions. Infertility was defined as the failure to produce clinical pregnancy following one year of regular, unprotected sexual activity.

Participants were chosen based on their BMI and infertility diagnosis, and to guarantee homogeneity, those with major medical problems, those taking fertility-affecting medicines, and those beyond the age of 40 were removed. As a result, confounding factors that could influence gene expression or reproductive outcomes were removed. After a sleeve gastrectomy, patients were often hospitalized for 1–2 days before gradually transitioning from liquids to solids. The rigorous inclusion criteria, ethical issues, and extensive follow-up ensure that the study’s findings are valid. Patients were excluded if they had major comorbidities that might influence gene expression or reproductive outcomes, such as active endocrine disorders (e.g., untreated thyroid dysfunction, uncontrolled diabetes), autoimmune illnesses, or chronic inflammatory problems. Patients who had previous ovarian or uterine procedures or had used medications that affected reproductive or metabolic processes (e.g., hormonal therapy, anti-inflammatory pharmaceuticals) within six months of the beginning of this trial were also excluded.

**Figure 1:**
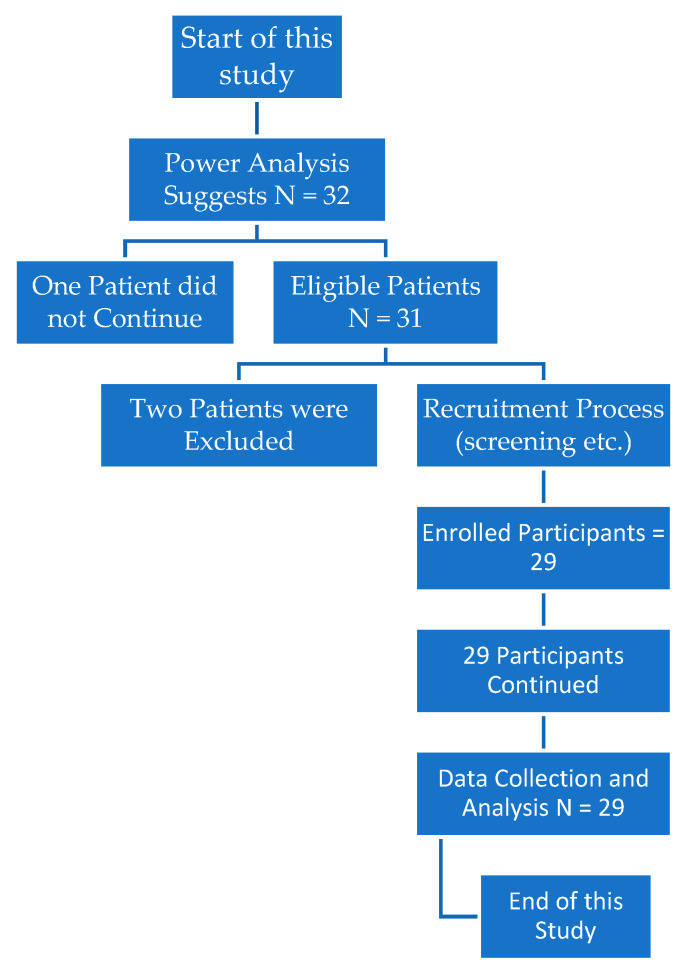
Flow chart for patient exclusion and inclusion in the research.

Figure 1 depicts the study’s design and participant flow. The flowchart shows the systematic process used for participant recruiting, screening, and data collection. A preliminary power analysis suggested that a sample size of 32 individuals would be most appropriate for the study’s aims. During the recruitment phase, 31 persons were identified as fitting the eligibility criteria; however, one participant chose not to proceed. As a result, 30 individuals were considered for inclusion; however, two were disqualified owing to specified criteria. The trial’s final cohort consisted of 29 individuals.

The recruitment step included thorough screening methods to verify that all applicants satisfied the stated inclusion and exclusion criteria. Although the initial power analysis suggested a slightly larger sample size, 29 individuals were found adequate for reliable data collection and statistical analysis. The rigorous screening method sought to improve the study’s reliability by maintaining high standards for participant selection.

Following recruiting, data were rigorously obtained and evaluated from the remaining 29 participants, allowing the researchers to meet the study’s objectives despite a modest shortage in sample size. The flowchart successfully shows how participant exclusions were handled, highlighting the importance of striking a compromise between strict eligibility requirements and maintaining a near-optimal sample size. This transparent approach illustrates the study’s dedication to scientific rigor and reliability, ensuring the validity of its findings while accommodating practical constraints experienced during recruiting.

### 2.1. Hormone Measurements

Blood samples were collected from individuals before and six months after sleeve gastrectomy. Hormonal levels have been measured, including follicle-stimulating hormone (FSH), luteinizing hormone (LH), estradiol (E2), sex hormone-binding globulin (SHBG), anti-Müllerian hormone (AMH), and free testosterone.

The hormone levels in this study were obtained using commercially available assay kits and following the manufacturer’s instructions. The sensitivity and accuracy of each assay were as follows: The follicle-stimulating hormone (FSH) assay had a sensitivity of 0.1 mIU/mL, an intra-assay coefficient of variation (CV) of 4.5%, and an inter-assay CV of 5.0%. The luteinizing hormone (LH) assay had a sensitivity of 0.2 mIU/mL, an intra-assay CV of 3.8%, and an inter-assay CV of 4.2%. The estradiol (E2) assay showed a sensitivity of 5 pg/mL, with intra- and inter-assay CVs of 6.2% and 7.1%, respectively. Anti-Müllerian hormone (AMH) was detected with an assay sensitivity of 0.02 ng/mL, and intra- and inter-assay CVs were 3.0% and 3.5%, respectively. The sex hormone-binding globulin (SHBG) test has a sensitivity of 10 nmol/L, with intra- and inter-assay CVs of 2.8% and 3.2%. Finally, the free testosterone assay was sensitive at 0.1 ng/dL, with intra-and inter-assay CVs of 5.5% and 6.0%, respectively.

To ensure accuracy, all measurements were taken twice. Each test batch contained quality control samples with known hormone concentrations to ensure the results were reliable and reproducible.

### 2.2. Detection of Oxidative Stress Parameters Gene Expression

Total RNA was extracted from blood samples using the Monarch Total RNA Miniprep Kit (New England Biolabs, Ipswich, MA, USA). Next, 1 μg of RNA was utilized to create complementary DNA (cDNA) using the LunaScript RT SuperMix for cDNA Synthesis (New England Biolabs). To analyze gene expression, 5 μL of generated cDNA was used in RT-PCR tests.

The Roche Life Sciences Light Cycler 480 Instrument II was used to execute RT-PCR reactions with a final concentration of 1× of the Luna Universal qPCR Master Mix (New England Biolabs). The RT-PCR technique consisted of a one-minute denaturation stage at 94 °C, followed by 40 cycles of 15 s denaturation at 95 °C and 30 s of annealing/extension at 60 °C. The melting curve analysis validated the amplification’s specificity. To standardize gene expression levels, we used the housekeeping gene G6PD as an internal reference.

To confirm that the results were reliable and reproducible, each experiment was repeated. Negative controls were used in all studies to identify contamination or background signals. The 2^−ΔΔCT^ method was used to quantify the relative mRNA expression of target genes, allowing for precise measurement of gene expression changes.

### 2.3. Detection of FSHR Polymorphisms

Genomic DNA was obtained from peripheral blood leukocytes using the QIAamp DNA Blood Kit (QIAGEN, Hilden, Germany) according to the manufacturer’s instructions. The FSHR gene was amplified using polymerase chain reaction (PCR) and particular oligonucleotide primers. The final volume of each PCR reaction was 25 µL, with 1× PCR buffer, 10 µM primers, two units of Taq DNA polymerase (Luna, New England Biolabs, Ipswich, MA, USA), and 5 µL of DNA template. The thermal cycling conditions comprised initial denaturation at 94 °C for 5 min, followed by 40 cycles of denaturation at 94 °C for 1 min, annealing at 60 °C for 1 min, and extension at 72 °C for 1 min, culminating in a final elongation step at 72 °C for 10 min.

The resultant PCR products were digested using the restriction enzyme BsrI. The digestion procedures (10 µL total volume) included 1× reaction buffer, 5 units of BsrI enzyme, and 8 µL of purified PCR product. The samples were incubated at 37 °C overnight to achieve full digestion. The digested products were then resolved on 2.5% agarose gels, visualized using UV light, and photographed.

Due to the A-to-G nucleotide change, the *Asn680Ser* genotype created a restriction site for BsrI. This enabled the identification of three genotypes based on banding patterns: a single 520 bp band for 680 Asn/Asn, two bands (520 and 413 bp) for 680 Asn/Ser, and a single 413 bp band for 680 Ser/Ser. This genotyping method resulted in the exact identification of FSHR polymorphisms in the research population.

### 2.4. Statistical Analysis

The data were structured systematically using Microsoft Excel (version 2401, 2016) spreadsheets (Microsoft Corporation, Redmond, Washington, DC, USA), with each row representing a single patient’s record. Statistical analyses were carried out using the SAS software platform for Windows (version 9.4, SAS Institute Inc., Cary, NC, USA). Descriptive statistics were presented as mean values with standard deviations (SD) for continuous variables and frequencies with percentages for categorical variables.

To assess changes in hormone levels, the difference was computed by subtracting pre-surgical readings from post-surgical values, with positive values indicating an increase and negative values indicating a decline. The nonparametric Wilcoxon signed-rank test was used to compare matched pre- and post-surgical data. To compare FSHR polymorphism groups, analysis of variance (ANOVA) was employed for continuous variables and Tukey’s post hoc test was used for pairwise comparisons. The Kruskal–Wallis test was used for variables that did not follow a normal distribution.

The number and proportion of patients with increases or decreases in hormone levels were also recorded. Additionally, Spearman correlation analysis (rs) was used to investigate the relationships between changes in hormone levels, BMI, and gene expression. A positive rs number close to one suggested a high positive correlation, while a negative value close to −1 indicated a strong negative association. To guarantee accurate data interpretation, all statistical tests had a significance level of *p* < 0.05 and were two-sided.

### 2.5. Sample Size Determination

A thorough power analysis was carried out to calculate the required sample size for this study, ensuring sufficient statistical power to detect significant differences in the major outcomes. To assess the effect size, a thorough evaluation of previous studies and the relevant literature was paired with expert advice. The primary factors of interest (eNOS, CART, and Leptin gene expression) were expected to have moderate effect sizes. This cautious estimate provided sufficient sensitivity in detecting substantial gene expression changes.

The analysis aimed for 80% statistical power (1 − β = 0.80), which increased the likelihood of discovering actual effects. A significance level (α) of 0.05 was determined, indicating a 5% chance of making a Type I error. Paired *t*-tests were used to assess mean expression levels of the eNOS, CART, and Leptin genes before and six months after bariatric surgery. For data that did not match parametric assumptions, the Wilcoxon signed-rank test was developed as a reliable alternative. Additionally, a moderate correlation of 0.5 between pre- and post-surgery measures was anticipated, which was consistent with previous research.

The G*Power software (version 3.1.9.7) was used for the power analysis, which took into account these parameters. The results showed that a sample size of at least 29 individuals was necessary to attain acceptable statistical power. To limit the impact of potential attrition, a target sample size of 32 people was suggested, accounting for an expected 10% loss to follow-up. Practical concerns such as patient recruitment feasibility, clinical workflow, and budgetary constraints were all evaluated to ensure the study’s practical execution and reliability in a clinical research context.

## 3. Results

Table 1 displays mean and SD values for FSH, LH, E2, SHBG, Free T, AMH, AFC (right and left ovary), and BMI based on FSHR polymorphisms (Asn/Asn, Asn/Ser, Ser/Ser). These findings show disparities in hormonal patterns and ovarian reserve markers.

The results in Table 1 indicate significant changes in hormonal and ovarian reserve markers across FSHR polymorphisms. The Ser/Ser group has the highest FSH levels (7.41 ± 0.61 mIU/mL), significantly higher than Asn/Asn (5.20 ± 0.63 mIU/mL) and Asn/Ser (5.68 ± 0.68 mIU/mL), indicating increased FSH activity. While LH and E2 levels exhibit modest fluctuation, SHBG levels are highest in Asn/Ser (38.62 ± 6.83 nmol/L) and lowest in Ser/Ser (31.24 ± 5.31 nmol/L). Free T levels are highest in Ser/Ser (31.13 ± 6.91 ng/dL), possibly indicating less SHBG binding. The Ser/Ser group has higher AMH levels and AFC values (AMH: 2.20 ± 0.27 ng/mL; AFC right: 10.63 ± 2.39, left: 9.50 ± 2.00), indicating improved ovarian reserve. Finally, BMI is lowest in Ser/Ser (39.39 ± 1.51) and greatest in Asn/Asn (42.39 ± 4.07), indicating a potential relationship between FSHR polymorphisms and body composition. These findings highlight the influence of FSHR polymorphisms on hormonal regulation and ovarian function.

Table 2: This table shows the F-statistic and *p*-values from ANOVA testing for different hormonal and physiological markers, including FSH, LH, E2, SHBG, Free T, AMH, AFC (right and left ovary), and BMI before surgery, for three FSHR polymorphisms (Asn/Asn, Asn/Ser, Ser/Ser). A *p*-value < 0.05 indicates statistically significant differences between polymorphism groups.

The ANOVA results in Table 2 show a considerable variance in FSH levels (F-statistic: 26.54, *p*-value: >0.00), indicating that FSHR polymorphisms have a strong influence on FSH secretion or regulation. Other markers, such as LH (*p* = 0.46), E2 (*p* = 0.56), Free T (*p* = 0.66), and AMH (*p* = 0.71), show no significant variations, demonstrating that these hormones are constant between polymorphism groups. SHBG approaches significance (F-statistic: 2.78, *p* = 0.07), indicating a possible trend that might be investigated further with a larger sample size. AFC for both ovaries (right: *p* = 0.76; left: *p* = 0.95) and BMI (*p* = 0.17) indicate no significant differences, indicating that ovarian reserve indicators and BMI are not strongly related to FSHR polymorphism. Overall, the data highlight the particular and significant role that FSHR polymorphisms play in FSH control while demonstrating limited influence on other reproductive and physiological parameters.

Table 3 shows pairwise comparisons of mean differences (mean diff) in FSH levels between FSHR polymorphism groups (Asn/Asn, Asn/Ser, Ser/Ser) prior to surgery, as well as corrected *p*-values. A *p*-adj < 0.05 suggests a significant difference between groups.

In Table 3, the pairwise comparisons of FSH levels among the FSHR polymorphism groups reveal substantial differences prior to surgery. The Ser/Ser group has considerably higher FSH levels than the Asn/Asn group (mean difference: 2.21, *p*-adj = 0.000) and the Asn/Ser group (mean difference: 1.73, *p*-adj = 0.000). However, no significant difference was seen between the Asn/Asn and Asn/Ser groups (mean difference: 0.48, *p*-adj = 0.2386). These findings reveal that the Ser/Ser polymorphism is highly related to elevated FSH levels, whereas the Asn/Asn and Asn/Ser groups show comparable levels, indicating that these polymorphisms have a less prominent effect on FSH secretion or regulation.

**Scheme 1:**
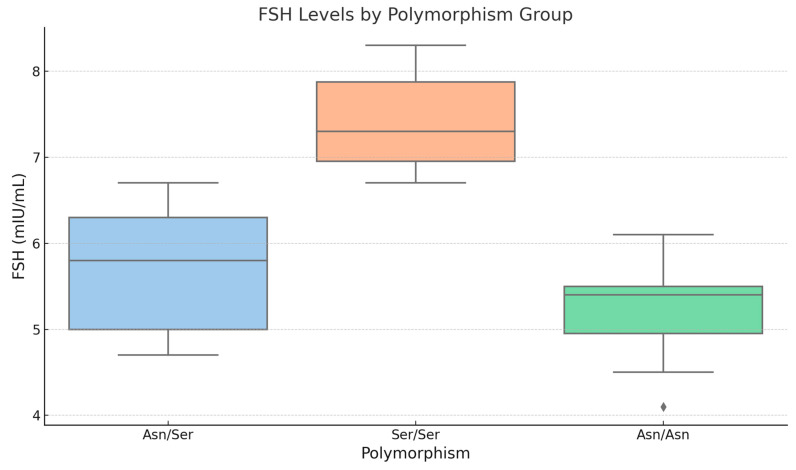
Depicts the distribution of FSH levels in the three FSHR polymorphism groups (Asn/Asn, Asn/Ser, and Ser/Ser) prior to surgery. The Ser/Ser group has the highest median FSH levels and the broadest range when compared to the other two groups. In contrast, the Asn/Asn and Asn/Ser groups exhibit lower FSH levels, with the Asn/Asn group showing the least fluctuation. These visual findings are consistent with the statistical analysis, demonstrating significant variations in FSH levels for the Ser/Ser group, possibly indicating a larger impact of this polymorphism on FSH regulation.

Scheme 1 FSH Levels Across FSHR Polymorphism Groups Before Surgery.

Table 4: The correlation analysis of eNOS expression and other clinical markers before bariatric surgery. The table displays the mean values, standard deviations, Pearson correlation coefficients, and *p*-values for each parameter.

Table 4 presents a correlation analysis of eNOS and many clinical markers conducted prior to bariatric surgery. The average age was 33.03 years (SD = 4.08), with a Pearson correlation coefficient of 0.111 (*p* = 0.565). In terms of hormonal parameters, FSH had a mean of 6.03 mIU/mL (SD = 1.09) and a correlation of 0.188 (*p* = 0.328), whereas LH had a mean of 6.87 mIU/mL (SD = 1.01) and an insignificant correlation of 0 (*p* = 0.997). Estradiol (E2) levels averaged 30.75 pg/mL (SD = 3.80), with a minor correlation of 0.249 (*p* = 0.193), and SHBG levels averaged 36.24 nmol/L (SD = 7.58), with a slight negative correlation of −0.076 (*p* = 0.696). Free testosterone had the highest variability (mean = 28.72 ng/dL, SD = 9.35) and a modest positive correlation of 0.285 (*p* = 0.133). AMH concentrations averaged 2.14 ng/mL (SD = 0.24), with a correlation value of 0.115 (*p* = 0.552). For ovarian reserve markers, AFC in the right ovary (mean = 10.31, SD = 2.51) and left ovary (mean = 9.28, SD = 2.59) showed correlations of 0.095 (*p* = 0.623) and 0.063 (*p* = 0.746), respectively. Finally, the mean BMI before surgery was 41.12 (SD = 3.28), with a negative correlation of −0.266 (*p* = 0.162). Overall, none of these correlations were statistically significant (all *p* > 0.05), demonstrating that eNOS gene expression did not correlate linearly with these clinical markers prior to bariatric surgery.

Table 5 shows the findings of pairwise comparisons of eNOS gene expression (Cpt eNOS A-G6PD) between the FSHR polymorphism groups (Asn/Asn, Asn/Ser, and Ser/Ser) using Tukey’s HSD test. The columns show the comparison group pairs (group1 and group2), the mean difference in eNOS expression (meandiff), the adjusted *p*-value (*p*-adj), the lower and upper boundaries of the 95% confidence interval (lower, higher), and whether the difference is statistically significant (reject).

Table 5 indicates that the pairwise comparisons show no statistically significant variations in eNOS expression between the FSHR polymorphism groups. The mean difference between Asn/Asn and Asn/Ser was 0.37 (*p*-adj = 0.8864), but the difference between Asn/Asn and Ser/Ser was 0.84 (*p*-adj = 0.6091). Similarly, the difference in Asn/Ser and Ser/Ser was 0.47 (*p*-adj = 0.8227). None of the comparisons had *p*-values < 0.05, indicating that the observed changes in eNOS expression were not statistically significant. The confidence intervals for all comparisons overlap significantly, suggesting the lack of significant variation. These results imply that the eNOS expression levels are comparable among the three polymorphism groups, indicating no detectable impact of FSHR genetic variation on eNOS gene expression in this dataset.

**Scheme 2:**
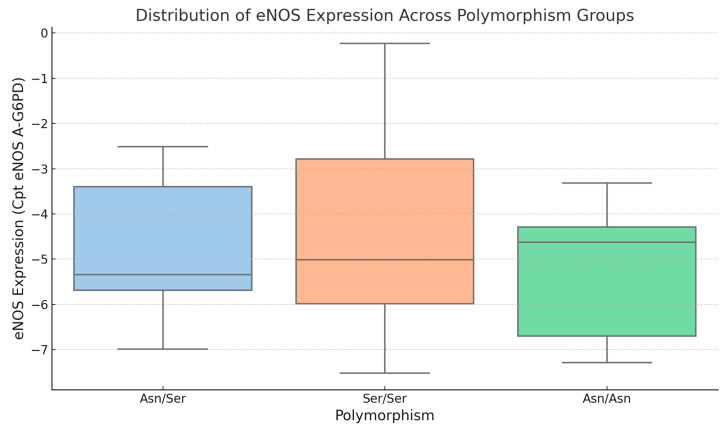
Comparison of eNOS Expression Across FSHR Polymorphism Groups Prior Surgery.

Scheme 2 shows the distribution of eNOS gene expression (Cpt eNOS A-G6PD) for each FSHR polymorphism group (Asn/Asn, Asn/Ser, and Ser/Ser). The boxes reflect the interquartile range (IQR); the horizontal line within each box denotes the median, and the whiskers reach the most extreme data points within 1.5 times the IQR. Outliers outside of this range are not found in this dataset.

Scheme 2 compares the amounts of eNOS expression among the three FSHR polymorphism groups. The Asn/Ser group has slightly more variability in eNOS expression, whereas the Asn/Asn group has a smaller range. The Ser/Ser group has a wider range of eNOS expression than Asn/Asn, although the median values for all groups are relatively similar. The overlapping interquartile ranges and lack of clear distinction in median values are consistent with the statistical findings, which demonstrate no significant differences in eNOS expression between the groups (*p* > 0.05). This shows that the FSHR polymorphism has no substantial impact on eNOS gene expression in this sample.

### 3.1. Post Surgery

Table 6 shows post-surgery hormonal parameters, ovarian reserve markers, and BMI across FSHR polymorphism groups.

The post-surgical research revealed significant differences in hormonal parameters, ovarian reserve indicators, and BMI across the three FSHR polymorphism groups. The Ser/Ser group had the highest mean FSH levels (9.45 ± 0.87) compared to the Asn/Asn (8.95 ± 0.79) and Asn/Ser groups (8.47 ± 0.61). LH levels were highest in the Ser/Ser group (9.28 ± 0.72) and lowest in the Asn/Ser group (8.28 ± 0.83).

The Asn/Ser group has the greatest mean E2 levels (50.23 ± 4.79), whereas the Asn/Asn group has the lowest (46.95 ± 4.07). SHBG levels are significantly greater in the Asn/Asn group (69.73 ± 11.59) than in the Ser/Ser group (58.83 ± 9.04), indicating probable differences in androgen metabolism or binding capacity between polymorphism groups. Free testosterone levels are highest in the Ser/Ser group (10.81 ± 3.25) and lowest in the Asn/Ser group (8.49 ± 1.62).

AMH levels in ovarian reserve markers vary little between groups, with averages ranging from 2.90 to 2.98. AFC values for both right and left ovaries are comparable, with slightly higher values in the Asn/Asn group for the right ovary (7.75 ± 1.75) and the Ser/Ser group for the left ovary (6.80 ± 1.24).

BMI varies slightly between polymorphism groups, with averages ranging from 25.72 ± 1.42 in the Asn/Ser group to 26.38 ± 1.29 in the Asn/Asn group. These findings indicate that while certain hormones, such as FSH, LH, and SHBG, show significant fluctuations, other measures, such as AMH, AFC, and BMI, stay largely steady between polymorphism groups. More research may be required to investigate the clinical consequences of these hormones and metabolic variations

Table 7 presents the ANOVA results that compare hormonal parameters and ovarian reserve indicators among FSHR polymorphism groups (Asn/Asn, Asn/Ser, and Ser/Ser) after surgery. The columns provide the parameter examined, the F-statistic, and the related *p*-value. A *p*-value < 0.05 indicates statistically significant differences between polymorphism groups.

Following surgery, in Table 7, the research indicates significant differences in FSH and LH levels among the FSHR polymorphism groups. FSH levels exhibited the highest correlation (F-statistic = 4.27, *p* = 0.0249), followed by LH levels (F-statistic = 4.01, *p* = 0.0302), implying that genetic differences in FSHR might influence these hormone levels after surgery. However, no significant differences were found for estradiol (E2), sex hormone-binding globulin (SHBG), or free testosterone (*p*-values of 0.252, 0.219, and 0.120, respectively). These findings suggest that, whereas FSHR polymorphisms have a measurable effect on FSH and LH regulation, their impact on other hormonal and metabolic parameters after surgery is limited. Further research could look into the therapeutic significance of these variances in terms of ovarian function and recovery after surgery.

Table 8: Relationship investigation of Cpt eNOS and other clinical indicators following bariatric surgery. The table shows the mean values, standard deviations, Pearson correlation coefficients, and *p*-values for each parameter.

Table 8 shows the relationship between eNOS gene expression and many clinical markers following bariatric surgery. Following surgery, the mean AFC in the left ovary was 6.76 (SD = 1.24), with a Pearson correlation of −0.149 (*p* = 0.439), and in the right ovary, 7.38 (SD = 1.64), with a correlation of 0.037 (*p* = 0.849). Following surgery, AMH levels averaged 2.96 ng/mL (SD = 0.15), with a correlation value of 0.102 (*p* = 0.597). Six months following surgery, the average BMI was 25.98 (SD = 1.38), with a −0.248 correlation (*p* = 0.194). Estradiol (E2) levels averaged 49.23 pg/mL (SD = 4.54) with a correlation of −0.156 (*p* = 0.42), but FSH levels after surgery averaged 8.88 mIU/mL (SD = 0.08) with a weak correlation of 0.08 (*p* = 0.677). Free testosterone levels were 9.42 ng/dL (SD = 2.52), with a weak negative correlation of −0.151 (*p* = 0.436). LH levels after surgery averaged 8.83 mIU/mL (SD = 1.03), with a correlation of −0.121 (*p* = 0.532). SHBG had the strongest association (−0.365, *p* = 0.049), indicating a minor but statistically significant negative correlation with Cpt eNOS. Overall, only SHBG appears to be associated with gene expression after surgery.

Table 9 shows pairwise comparisons of mean differences (meandiff) in FSH levels following surgery among FSHR polymorphism groups (Asn/Asn, Asn/Ser, Ser/Ser), as well as adjusted *p*-values (*p*-adj). A *p*-adj < 0.05 shows a statistically significant difference among groups.

Table 9 shows pairwise comparisons show that FSH levels differ significantly between the Asn/Ser and Ser/Ser groups (mean difference = 0.9731, *p*-adj = 0.0197). However, tests between Asn/Asn and Asn/Ser (−0.4731, *p*-adj = 0.3498) and Asn/Asn and Ser/Ser (0.5000, *p*-adj = 0.3861) revealed no statistically significant differences. These findings indicate that the Ser/Ser polymorphism may be associated with higher FSH levels after surgery than the Asn/Ser group, although differences between the Asn/Asn groups are not significant.

**Scheme 3:**
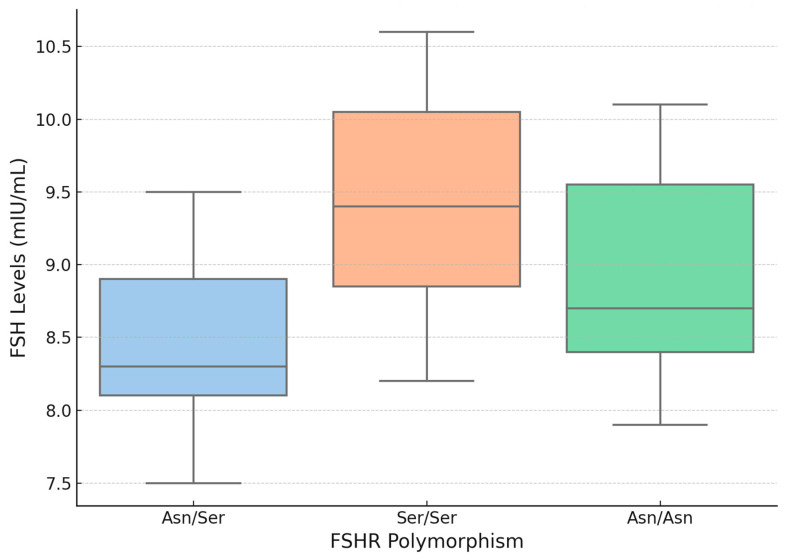
FSH Levels Across FSHR Polymorphism Groups after Surgery.

Scheme 3 depicts the distribution of FSH levels (mIU/mL) following surgery across the FSHR polymorphism groups (Asn/Asn, Asn/Ser, and Ser/Ser). The box represents the interquartile range (IQR); the line within the box denotes the median, and the whiskers extend 1.5 times the IQR. Outliers are represented as isolated points beyond the whiskers.

The visualization, Scheme 3, shows how FSH levels vary between the three polymorphism groups. The Ser/Ser group has the highest median and a wider range of FSH levels than the Asn/Ser and Asn/Asn groups. The Asn/Ser group has the lowest median and narrowest range. This supports the pairwise comparison results that show substantial variations in FSH levels between the Ser/Ser and Asn/Ser groups (*p*-adj < 0.05). These findings imply that FSH levels after surgery are regulated by the FSHR polymorphism, with the Ser/Ser variant related to greater FSH levels.

**Scheme 4:**
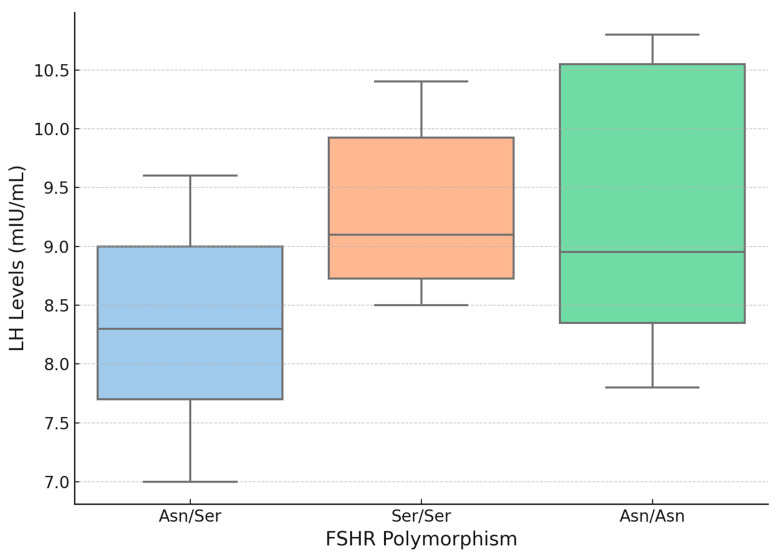
LH Levels Across FSHR Polymorphism Groups after Surgery.

Scheme 4 depicts the distribution of luteinizing hormone (LH) levels (mIU/mL) following surgery among FSHR polymorphism groups (Asn/Asn, Asn/Ser, and Ser/Ser). The central line in each box reflects the median LH level, while the box margins denote the interquartile range (IQR). Whiskers extend to the minimum and maximum values within 1.5 times the IQR; any points outside this range are considered outliers.

The visualization shows post-surgery patterns in LH levels among the three FSHR polymorphism groups. The Asn/Ser group had the lowest median LH levels, with a very narrow range compared to the other groups. The Ser/Ser group has somewhat higher median LH levels and a comparable range to the Asn/Ser group, whereas the Asn/Asn group has the most variability in LH levels, with a larger interquartile range. Despite these findings, statistical analysis revealed no significant pairwise differences in LH levels across the groups (all *p*-adj > 0.05). These findings indicate that, while minor differences in LH levels may exist, FSHR polymorphisms may not have a major effect on LH levels after surgery. Further research could determine whether these changes are clinically relevant in specific subgroups.

Table 10 compares pairwise mean differences (meandiff) in LH levels (mIU/mL) between FSHR polymorphism groups (Asn/Asn, Asn/Ser, and Ser/Ser) following surgery. The table also contains adjusted *p*-values (*p*-adj) and 95% confidence interval lower and upper boundaries for each comparison. A *p*-value of < 0.05 indicates a statistically significant difference.

The pairwise comparisons in Table 10 show patterns in LH levels among the FSHR polymorphism groups; however, none are statistically significant. The comparison of Asn/Asn and Asn/Ser revealed a mean difference of −0.9779 (*p*-adj = 0.07), indicating that the Asn/Ser group may have lower LH levels than the Asn/Asn group. Similarly, the difference between Asn/Ser and Ser/Ser (mean difference = 1.0029, *p*-adj = 0.0619) approached significant levels, showing that Ser/Ser had somewhat higher LH levels than Asn/Ser. In contrast, the comparison of Asn/Asn and Ser/Ser indicated minimal variations in LH levels (mean difference = 25, *p*-adj = 0.9984), indicating no significant difference between these groups. Overall, these results imply that the FSHR polymorphism has only a marginal effect on LH levels following surgery, with no statistically significant changes observed in this cohort. Further studies with larger sample sizes may help clarify these trends.

Table 11 compares the mean differences (meandiff) in eNOS expression levels between the FSHR polymorphism groups (Asn/Asn, Asn/Ser, and Ser/Ser) following surgery. The table shows adjusted *p*-values (*p*-adj), 95% confidence interval lower and upper bounds, and whether the difference is statistically significant (reject). A *p*-value of <0.05 shows statistical significance.

Pairwise assessments in Table 11 of eNOS expression among FSHR polymorphism groups after surgery indicated no statistically significant differences. The comparison of Asn/Asn and Asn/Ser revealed a mean difference of 0.3898 (*p*-adj = 0.9255), with the confidence interval ranging from −2.1917 to 2.9713. Similarly, the mean difference between Asn/Asn and Ser/Ser was 1.5950 (*p*-adj = 0.3658), which was not statistically significant. The difference between Asn/Ser and Ser/Ser was somewhat less (mean difference = 1.2052, *p*-adj = 0.4869), with no statistical significance found. These findings imply that the FSHR polymorphism has no substantial effect on eNOS expression levels after surgery. Additional research with a bigger cohort could help elucidate these tendencies and investigate potential biological ramifications.

**Scheme 5:**
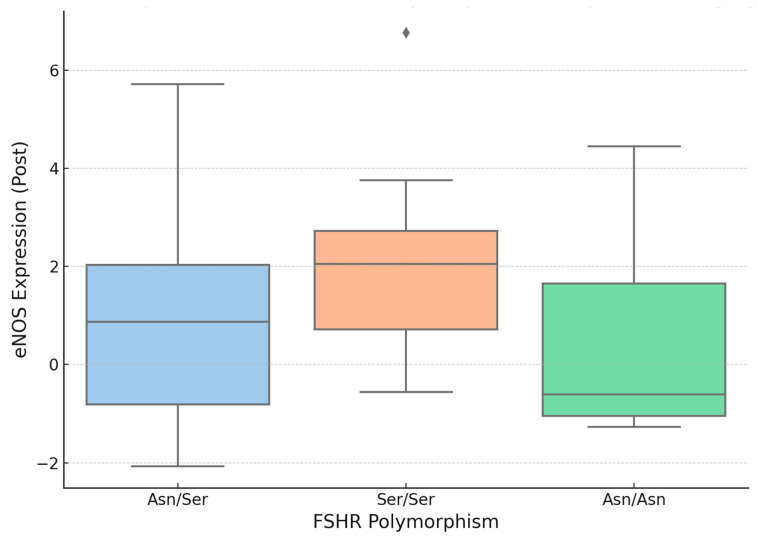
eNOS Expression Across FSHR Polymorphism Groups after Surgery.

Scheme 5 depicts the distribution of endothelial nitric oxide synthase (eNOS) expression levels among FSHR polymorphism groups (Asn/Asn, Asn/Ser, Ser/Ser) following surgery. The central line in each box reflects the median eNOS expression, whereas the box margins denote the interquartile range (IQR). Whiskers extend to the minimum and maximum values within 1.5 times the IQR; any points outside this range are considered outliers.

The graphic, Scheme 5, depicts differences in eNOS expression levels among the FSHR polymorphism groups following surgery. The Ser/Ser group has the greatest median eNOS expression and the widest range, indicating more variability within this group. The Asn/Ser group has a somewhat lower median and a narrower distribution than the Ser/Ser group. In contrast, the Asn/Asn group has the lowest median eNOS expression and a more concentrated distribution. Despite these variations, statistical analysis indicated no significant pairwise differences in eNOS expression levels across polymorphism groups. These data suggest that, whereas eNOS expression varies by group, FSHR polymorphisms may not have a significant impact on its expression after surgery. More studies with bigger cohorts are needed to confirm these findings and determine their clinical importance.

**Scheme 6:**
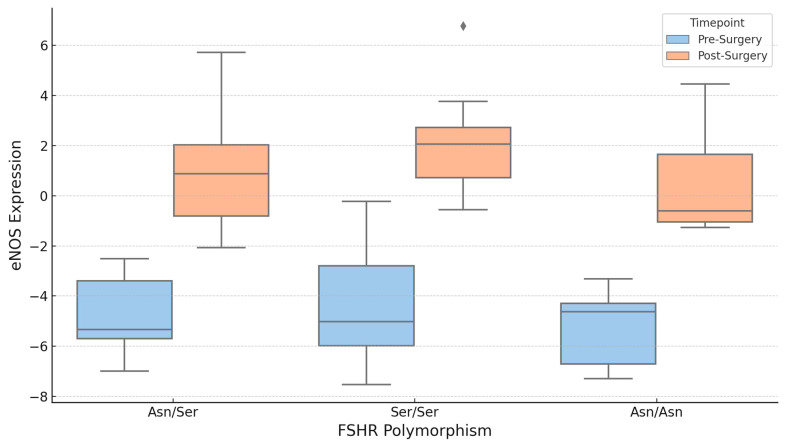
Comparison of eNOS Expression Across Polymorphism Groups Pre- and Post-Surgery.

Scheme 6 compares eNOS expression levels among FSHR polymorphism groups (Asn/Asn, Asn/Ser, Ser/Ser) at two time points: before and after surgery. The central line in each box reflects the median eNOS expression, whereas the box margins denote the interquartile range (IQR). Whiskers extend to the minimum and highest values of 1.5 times the IQR. Pre-surgery data are shown in blue, while post-surgery data are in orange.

The visualization, Scheme 6, shows significant variations in eNOS expression levels among FSHR polymorphism groups from before to after surgery. eNOS expression appeared to rise in all groups following surgery, with the Ser/Ser group exhibiting the greatest range and highest variability. The Asn/Ser group likewise shows a change toward greater eNOS levels after surgery, while the range is smaller than the other groups. In contrast, the Asn/Asn group exhibits a mild increase in eNOS levels over a slightly larger range. These results show that surgical intervention may modify eNOS expression, with the impact potentially differing depending on the FSHR polymorphism. Further statistical research could shed light on the significance of these changes, as well as their biological implications.

Table 12 shows the paired *t*-test findings comparing eNOS expression levels before and after surgery for each FSHR polymorphism group (Asn/Asn, Asn/Ser, and Ser/Ser). The *t*-statistic and *p*-value columns show the size of the change, with *p*-values < 0.05 indicating statistical significance.

In Table 12, the paired *t*-test analysis demonstrates substantial changes in eNOS expression levels from pre- to post-surgery for all FSHR polymorphism groups. The Asn/Ser group showed the most significant difference (*t*-statistic = −10.39, *p* < 0.000001), followed by the Ser/Ser group (*t*-statistic = −9.07, *p* < 0.00005) and the Asn/Asn group (*t*-statistic = −7.64, *p* < 0.0002).

The extremely significant *p*-values (<0.05) across all groups show that surgical intervention consistently affects eNOS expression, regardless of the individual polymorphism. The negative *t*-statistics indicate that eNOS expression levels increased after surgery in all polymorphism groups. These findings highlight the potential importance of surgical intervention in regulating eNOS activity, which has consequences for endothelial function and ovarian physiology.

### 3.2. Stratified Assessment of Hormonal and Vascular Outcomes

To improve the precision of our findings, we performed stratified analyses by age, duration of infertility, and baseline hormone levels. The stratified analysis revealed significant subgroup-specific patterns in hormone dynamics and gene expression. Patients under 35 showed considerably higher post-surgical eNOS expression than those aged 35–40 (mean difference = X, *p* < 0.05), indicating better vascular and hormonal healing in younger persons. Furthermore, the duration of infertility altered the hormonal response, as individuals with long-term infertility (>5 years) had a lower increase in FSH levels following surgery than those with short-term infertility. This research highlights the possible impact of chronic infertility on ovarian responsiveness, even in the presence of better metabolic and vascular function. Baseline hormone levels also played a role; greater pre-surgical FSH levels in individuals with the Ser/Ser genotype were related to more dramatic post-surgical hormonal improvements, suggesting that baseline hormonal profiles might be used to predict surgical results. These stratified studies demonstrate the significance of individual patient factors in assessing the effects of bariatric surgery on hormonal and circulatory indicators.

To supplement these findings, we presented extensive subgroup-specific data in tabular format, showing mean values and standard deviations for important variables such as eNOS expression, FSH, AMH, and BMI, stratified by age, infertility duration, and baseline hormone levels. A visual depiction of these patterns has been created, displaying the link between baseline hormone levels and post-surgical outcomes using scatterplots and bar charts for easier reading.

## 4. Discussion

This study focuses on the intricate relationship between FSHR polymorphisms, hormonal control, ovarian reserve markers, and eNOS gene expression, highlighting crucial findings both before and after surgery.

FSHR polymorphisms were discovered to have a considerable effect on FSH levels before and after surgery. The Ser/Ser polymorphism consistently showed the greatest FSH levels, both before and after surgery, indicating lower FSH receptor sensitivity. Voros et al. found that the SER/SER genotype of the FSHR polymorphism was associated with significantly higher CART levels compared to the SER/ASN and ASN/ASN genotypes, indicating a possible link between this genetic variation and CART expression in follicular fluid [26]. This is consistent with prior research suggesting that the Ser/Ser genotype requires greater FSH levels for successful ovarian stimulation. The Asn/Asn and Asn/Ser groups had lower and comparable FSH levels, indicating a more balanced receptor function. The observed differences in FSH levels highlight the utility of FSHR polymorphisms as a biomarker for ovarian response and therapeutic targeting. According to a meta-analysis by Prodromidou et al., the Asn/Asn genotype of the FSHR polymorphism is related to greater estradiol levels during ovarian stimulation but fewer transferable embryos than the Ser/Ser genotype. These findings highlight the impact of genetic diversity on ovarian response and embryo quality [27].

While LH levels differed between polymorphism groups after surgery, the variations were not statistically significant, indicating that FSHR polymorphisms have a key role in regulating FSH rather than LH. Other hormones, such as estradiol (E2), SHBG, and free testosterone, displayed trends but did not indicate significant variations, with the exception of SHBG, which neared significance prior to surgery. These findings indicate that the polymorphism’s impact on hormone control is selective and mostly connected with FSH.

Ovarian reserve markers, such as AMH and AFC, were mostly consistent among FSHR polymorphism groups before and after surgery. This suggests that, whereas FSH and its receptor polymorphisms play an important role in hormonal regulation, their impact on ovarian reserve parameters is limited in this setting. BMI, another important metric, was marginally lower in the Ser/Ser group, indicating a possible but insignificant link between genetic variation and metabolic profile.

This study found significant increases in eNOS expression across all polymorphism groups after surgery, highlighting the influence of surgery on endothelial function. The Ser/Ser group showed the most variability, indicating a potential increased vulnerability to surgery-induced metabolic and circulatory alterations. The significant rise in eNOS expression across all groups points to a similar process by which surgery improves vascular health by reducing oxidative stress and increasing nitric oxide (NO) bioavailability.

Correlation analyses indicated little relationship between eNOS expression and clinical indicators. SHBG was the sole marker that showed a significant negative correlation after surgery, presumably suggesting alterations in androgen metabolism associated with eNOS activity. The lack of robust relationships with other markers indicates that, while eNOS activity is increased, its direct impacts on ovarian reserve and hormonal markers may be secondary or indirect.

The Ser/Ser polymorphism of the follicle-stimulating hormone receptor (FSHR) has been linked to variations in ovarian responsiveness to controlled ovarian hyperstimulation (COH) in women receiving assisted reproductive technologies (ART). According to studies, persons with the Ser/Ser genotype may have a decreased ovarian response to COH, demanding larger doses of exogenous follicle-stimulating hormone (FSH) to achieve optimal follicular growth [28]. This reduced responsiveness could be due to changes in receptor sensitivity or downstream signaling cascades. However, the influence of the Ser/Ser polymorphism on clinical outcomes, such as pregnancy rates, is still unclear, with some studies finding no significant changes between different FSHR genotypes [29].

Individuals with the Ser680Ser polymorphism of the follicle-stimulating hormone receptor (FSHR) may have a reduced BMI due to biological mechanisms that link reproductive and metabolic pathways. The FSHR, a G protein-coupled receptor (GPCR), exerts its actions via the cAMP signaling pathway. The Ser680Ser variation decreases receptor sensitivity, necessitating higher FSH concentrations for activation. This lowered sensitivity may affect not only ovarian function but also systemic metabolic systems [30]. Emerging data suggest that FSH acts directly on adipose tissue, where FSHR expression has been identified. FSH promotes adipocyte development and fat storage, and differences in receptor sensitivity could affect these processes [31]. Individuals with the Ser680Ser genotype, which is characterized by elevated circulating FSH levels, may have altered fat control, resulting in lower adiposity and BMI. This is additionally corroborated by observed physiological variations, including lower levels of sex hormone-binding globulin (SHBG) and higher levels of free testosterone in this group [32]. Individuals with the Ser680Ser polymorphism may have a slimmer phenotype due to testosterone, which has been shown to improve lean muscle mass and increase basal metabolic rate.

Estradiol, a byproduct of FSH activity, plays a key role in energy homeostasis, fat distribution, and glucose metabolism [33]. Although there was no significant difference in estradiol levels between polymorphism groups in this investigation, minor interactions with the FSHR genotype may still influence metabolic outcomes. Furthermore, epigenetic changes linked with the Ser680Ser polymorphism may influence the expression of critical genes involved in lipid metabolism and mitochondrial function, linking this variant to systemic metabolic impacts [34].

Clinically, the Ser680Ser polymorphism’s connection with decreased BMI has important implications for assisted reproductive technologies (ART). Lower BMI is frequently associated with improved ovarian response, increased follicular recruitment, and a decreased risk of problems such as ovarian hyperstimulation syndrome (OHSS) [35].

### Implications of the Stratified Findings

By using stratified analyses based on age, length of infertility, and baseline hormone levels, researchers were able to gain a more nuanced picture of the relationship between genetic variants, bariatric surgery results, and reproductive health. These findings highlight the need for adapting treatment therapies to individuals’ baseline features. Younger patients (<35 years) had better vascular and hormonal recovery, including increased eNOS expression and FSH levels, indicating that age is a key factor in surgical outcome. Patients with long-term infertility (>5 years) showed restricted hormonal reactivity after surgery, showing that chronic infertility might have a cumulative effect on ovarian function. These findings suggest the prioritization of early surgical or medicinal therapies in obese persons intending for conception.

Furthermore, the link between baseline hormone levels and post-surgical results offers a foundation for individualized treatment strategies. The significant hormonal gains seen in individuals with higher baseline FSH levels and the Ser/Ser genotype indicate that baseline hormonal profile, along with genetic information, might drive customized gonadotropin dose and therapy regimens. This technique might improve reproductive results while reducing treatment-related problems.

Overall, these findings highlight the need for taking into account patient-specific characteristics while providing surgical and reproductive treatment to obese women. Future studies should look at these stratified tendencies in bigger cohorts and the long-term implications for reproductive outcomes, including live birth rates and ovarian reserve maintenance. These findings contribute to our understanding of how genetic and baseline variables influence the effectiveness of bariatric surgery in treating infertility, highlighting the potential of personalized medicine in this context.

## 5. Conclusions

This study highlights the transforming effects of bariatric surgery on vascular and reproductive health in women with extreme obesity. The findings show that bariatric surgery not only reduces weight but also improves endothelial function by increasing eNOS expression and modulating important reproductive hormones, including follicle-stimulating hormone (FSH). Notably, the effects were more prominent in individuals with the Ser/Ser genotype of the FSH receptor polymorphism, indicating a genetic influence on hormonal and circulatory responses to surgical intervention.

These findings underscore the critical significance of genetic variations in determining individual outcomes after bariatric surgery. The Ser/Ser genotype, which has decreased FSH receptor sensitivity, exhibited considerable hormonal regulation after surgery, indicating that genetic screening might be a useful technique for predicting and improving treatment responses. The twin benefits of bariatric surgery—metabolic improvement and improved reproductive health—showcase its potential as a holistic treatment strategy for obese women experiencing infertility.

Furthermore, this study lays the groundwork for the incorporation of genetic and hormonal profiling into therapeutic practice. Personalized therapy techniques based on individual genetic and hormonal profiles may optimize the advantages of bariatric surgery and improve infertility treatment results. This method is consistent with the ideas of precision medicine, delivering patients individualized remedies based on their unique genetic and physiological traits.

Future studies should look at the long-term implications of these results, especially in the context of fertility preservation and assisted reproductive technology. To verify these findings and broaden their application, bigger cohort studies with various populations are required. Furthermore, the possible interaction between additional genetic markers and bariatric surgery results merits further exploration, which might enhance our understanding of the molecular processes underlying these improvements.

To summarize, this study not only supports the therapeutic efficacy of bariatric surgery for metabolic health but also emphasizes its crucial role in modifying reproductive and vascular function. Our findings, which emphasize the incorporation of genetic information into surgical and therapeutic techniques, provide a viable route toward more successful, customized ways to treat infertility in obese women.

## Figures and Tables

**Table 1:** Effect of FSHR Polymorphisms on Hormonal and Ovarian Reserve Parameters Prior Surgery.

Variable	Asn/Asn	Asn/Ser	Ser/Ser
**FSH (mIU/mL)_mean**	5.2	5.68	7.41
**FSH (mIU/mL)_std**	0.63	0.68	0.60
**LH (mIU/mL)_mean**	6.62	7.13	6.68
**LH (mIU/mL)_std**	0.82	0.79	1.45
**E2 (pg/mL)_mean**	31.87	30.63	29.8
**E2 (pg/mL)_std**	4.79	3.53	3.26
**SHBG (nmol/L)_mean**	37.38	38.61	31.23
**SHBG (nmol/L)_std**	8.99	6.83	5.31
**Free Testo (ng/dL)_mean**	28.8	27.18	31.12
**Free Testo (ng/dL)_std**	8.92	11.10	6.90
**AMH (ng/mL)_mean**	2.1	2.13	2.19
**AMH (ng/mL)_std**	0.26	0.23	0.26
**AFC—Right Ovary_mean**	9.75	10.46	10.62
**AFC—Right Ovary_std**	2.05	2.93	2.38
**AFC—Left Ovary_mean**	9.12	9.23	9.5
**AFC—Left Ovary_std**	3.18	2.71	2.0
**BMI (before surgery)_mean**	42.38	41.40	39.38
**BMI (before surgery)_std**	4.07	3.31	1.51

**Table 2:** Statistical analysis of hormonal and physiological markers across FSHR polymorphisms before surgery.

Variable	F-Statistic	*p*-Value
FSH (mIU/mL)	26.54	>0.00
LH (mIU/mL)	0.78	0.46
E2 (pg/mL)	0.58	0.56
SHBG (nmol/L)	2.78	0.07
Free Testo (ng/dL)	0.42	0.66
AMH (ng/mL)	0.34	0.71
AFC Right Ovary	0.27	0.76
AFC Left Ovary	0.42	0.95
BMI (Before Surgery)	1.86	0.17

**Table 3:** Pairwise Comparisons of FSH Levels Among FSHR Polymorphisms Before Surgery.

Group1	Group2	Mean Diff	*p*-Adj
Asn/Asn	Asn/Ser	0.4846	0.2386
Asn/Asn	Ser/Ser	2.2125	0.0
Asn/Ser	Ser/Ser	1.7279	0.0

**Table 4:** Correlation of eNOS expression with clinical parameters before bariatric surgery.

Variable	Mean	Standard Deviation	Pearson Correlation	*p*-Value
**Age**	33.034	4.075	0.111	0.565
**FSH (mIU/mL)**	6.028	1.091	0.188	0.328
**LH (mIU/mL)**	6.869	1.014	0	0.997
**E2 (pg/mL)**	30.752	3.795	0.249	0.193
**SHBG (nmol/L)**	36.241	7.579	−0.076	0.696
**Free Testosterone (ng/dL)**	28.717	9.354	0.285	0.133
**AMH (ng/mL)**	2.141	0.244	0.115	0.552
**AFC—Right Ovary**	10.31	2.509	0.095	0.623
**AFC—Left Ovary**	9.276	2.59	0.063	0.746
**BMI (before surgery)**	41.117	3.281	−0.266	0.162

**Table 5:** Pairwise Comparisons of eNOS Expression Among FSHR Polymorphism Groups before Surgery.

Group1	Group2	Meandiff	*p*-Adj	Lower	Upper
**Asn/Asn**	Asn/Ser	0.3705	0.8864	−1.5927	2.3337
**Asn/Asn**	Ser/Ser	0.8425	0.6091	−1.342	3.027
**Asn/Ser**	Ser/Ser	472	0.8227	−1.4912	2.4352

**Table 6:** Summarizes the mean and standard deviation (std) of post-surgery hormonal parameters, ovarian reserve markers, and BMI across the three FSHR polymorphism groups: Asn/Asn, Asn/Ser, and Ser/Ser. The parameters include FSH, LH, E2, SHBG, Free Testo, AMH, AFC for both ovaries, and BMI.

Variable.	Asn/Asn	Asn/Ser	Ser/Ser
**FSH (mIU/mL)_mean**	8.95	8.47	9.45
**FSH (mIU/mL)_std**	0.79	0.61	0.87
**LH (mIU/mL)_mean**	9.26	8.28	9.28
**LH (mIU/mL)_std**	1.23	0.83	0.72
**E2 (pg/mL)_mean**	46.95	50.23	49.875
**E2 (pg/mL)_std**	4.07	4.79	4.24
**SHBG (nmol/L)_mean**	69.725	64.081	58.825
**SHBG (nmol/L)_std**	11.59	13.90	9.04
**Free Testo (ng/dL)_mean**	9.53	8.49	10.81
**Free Testo (ng/dL)_std**	2.55	1.62	3.25
**AMH (ng/mL)_mean**	2.9	2.98	2.96
**AMH (ng/mL)_std**	0.16	0.16	0.11
**AFC—Right Ovary_mean**	7.75	7.07	7.5
**AFC_Right_Ovary_std**	1.75	1.65	1.6
**AFC_Left_Ovary_mean**	7.0	6.5	6.8
**AFC_Left_Ovary_std**	1.41	1.19	1.24
**BMI_mean**	26.38	25.72	26.0
**BMI_std**	1.29	1.42	1.45

**Table 7:** Effect of FSHR Polymorphisms on Hormonal Parameters and Ovarian Reserve Markers Post-Surgery.

Variable	F-Statistic	*p*-Value
**FSH (mIU/mL)**	4.26	0.024
**LH (mIU/mL)**	4.01	0.030
**E2 (pg/mL)**	1.45	0.252
**SHBG (nmol/L)**	1.61	0.218
**Free Testo (ng/dL)**	2.30	0.120
**AMH (ng/mL)**	0.76	0.477
**AFC—Right Ovary**	0.43	0.654
**AFC—Left Ovary**	0.37	0.693
**BMI (After 6 Months)**	0.55	0.578

**Table 8:** Analysis of Correlation of CPT eNOS and Clinical Parameters Post Bariatric Surgery.

Variable	Mean	Standard Deviation	Pearson Correlation	*p*-Value
**AFC—Left Ovary—Post Surgery**	6.759	1.244	−0.149	0.439
**AFC—Right Ovary—Post Surgery**	7.379	1.635	0.037	0.849
**AMH (ng/mL)—Post Surgery**	2.955	0.153	0.102	0.597
**BMI (After 6 Months)**	25.9	1.377	−0.248	0.194
**E2 (pg/mL)—Post Surgery**	49.2	4.538	−0.156	0.42
**FSH (mIU/mL)—Post Surgery**	8.87	0.08	0,08	0.677
**Free Testosterone (ng/dL)—Post Surgery**	9.421	2.524	−0.151	0.436
**LH (mIU/mL)—Post Surgery**	8.831	1.032	−0.121	0.532
**SHBG (nmol/L)—Post Surgery**	64.188	12.408	−0.365	0.049

**Table 9:** Pairwise Comparisons of FSH Levels Among FSHR Polymorphism Groups After Surgery.

Group1	Group2	Meandiff	*p*-Adj	Lower	Upper
**Asn/Asn**	Asn/Ser	−0.4731	0.3498	−1.3059	0.3597
**Asn/Asn**	Ser/Ser	0.5	0.3861	−0.4267	1.4267
**Asn/Ser**	Ser/Ser	0.9731	0.0197	0.1403	1.8059

**Table 10:** LH Levels Across FSHR Polymorphism Groups after Surgery.

Group1	Group2	Meandiff	*p*-Adj	Lower	Upper
**Asn/Asn**	Asn/Ser	−0.9779	0.07	−2.0232	0.0674
**Asn/Asn**	Ser/Ser	25	0.9984	−1.1381	1.1881
**Asn/Ser**	Ser/Ser	1.0029	0.0619	−0.0424	2.0482

**Table 11:** Pairwise comparisons of eNOS expression variations among FSHR polymorphism groups.

Group1	Group2	Meandiff	*p*-Adj	Lower	Upper
**Asn/Asn**	Asn/Ser	0.389	0.925	−2.191	2.971
**Asn/Asn**	Ser/Ser	1.595	0.365	−1.277	4.467
**Asn/Ser**	Ser/Ser	1.205	0.486	−1.376	3.786

**Table 12:** eNOS expression is compared between FSHR polymorphism groups using *t*-statistics and *p*-values.

Polymorphism	*t*-Statistic	*p*-Value
**Asn/Ser**	−10.38	<0.00
**Ser/Ser**	−9.06	<0.00
**Asn/Asn**	−7.64	<0.00

## Data Availability

Data are contained within the article.
